# Measurement of the Turbopause Level and Turbulent Velocity in the Lower Ionosphere Based on the Creation of Artificial Periodic Irregularities in the Ionospheric Plasma

**DOI:** 10.3390/s26185896

**Published:** 2026-09-17

**Authors:** Nataliya V. Bakhmetieva, Ilia N. Zhemyakov, Elena E. Kalinina

**Affiliations:** Radiophysical Research Institute, Nizhny Novgorod State University, 25/12a Bol’shaya Pecherskaya Str., 603950 Nizhny Novgorod, Russia

**Keywords:** ionosphere, API technique, artificial periodic irregularities, relaxation time, turbopause level, turbulent velocity, regular motion, atmospheric waves

## Abstract

**Highlights:**

**What are the main findings?**

- A new method based on the creation of artificial periodic irregularities in the ionospheric plasma was applied to study atmospheric turbulence in the lower ionosphere.
- The turbopause height and turbulent velocity were determined in different space weather conditions.

**What are the implications of the main findings?**

- The results show that the applied method gives realistic values of turbulent parameters.
- Internal gravity waves make a significant contribution to variations in the turbopause level and turbulent velocity.

**Abstract:**

We have developed a new method for remote sensing of the ionosphere and neutral atmosphere based on the creation of artificial periodic irregularities (APIs) in ionospheric plasma. This method is used, in particular, to study atmospheric turbulence, which can affect GNSS signal delays by causing disturbances in the radio wave propagation medium. We applied the API method to determine the turbopause height and turbulent velocity at altitudes of 60–130 km in the lower ionosphere. Turbulence is one of the most important phenomena in the Earth’s atmosphere. This article presents the results of turbulence parameter measurements based on the resonant scattering of APIs. These irregularities are created in the ionosphere by powerful high-frequency radio waves. The altitude profile of turbulent velocity is obtained from measurements of the relaxation time of the API scattered signal after the end of the powerful radio emission on the ionosphere. The turbopause level is defined as the altitude at which turbulent mixing of atmospheric gases gives way to diffusive separation. During the transition from diffusive separation to turbulent mixing (below the turbopause level), irregularities decay more rapidly than during ambipolar diffusion, and their relaxation time is determined predominantly by turbulent diffusion. This fact allows us to determine the velocity of turbulent motion as a function of the diffusion time and the measured relaxation time of the scattered signal. The turbopause level is determined from the altitude profile of the relaxation time. Measurements were carried out using the SURA heating facility (56.15° N, 46.11° E). The altitude resolution of the API method is 0.15 km–1 km with a time resolution of 15 s, which allows us to study both fast and slow processes. According to our data, the turbopause level varied in the altitude range from 85 km to 110 km. Significant variability in the turbopause level was observed during the day and from day to day. The minimum turbopause level was located at mesospheric altitudes. The average turbulent velocity over a 5 min interval varied from near zero at the turbopause altitude to 5–6 m/s below it. Such high velocities were typically observed during natural ionospheric disturbances. The average velocity of regular vertical plasma motion, measured using the API method, reached 10 m/s and varied with altitude and direction. Temporal variations in turbulent parameters were observed, with periods ranging from 15 min to several hours. The effect of atmospheric wave propagation on the characteristics of scattered signals and environmental parameters was confirmed.

## 1. Introduction

Turbulence is one of the most important phenomena in the Earth’s atmosphere. It makes a significant contribution to atmospheric dynamics. We study atmospheric turbulence in the 60–130 km altitude range. This range is the lower ionosphere, which includes the mesosphere and lower thermosphere (MLT). This transition region between the lower and upper atmosphere facilitates interaction between the troposphere, which shapes weather and climate, and the thermosphere, which is regulated by solar activity. The lower ionosphere and MLT region are characterized by developed dynamics, which include variations in neutral temperature and density, electron concentration, and horizontal and vertical motions. Atmospheric waves propagate in this altitude range, and turbulent phenomena promote mixing of air components, ensuring a constant composition up to the turbopause altitude, at which turbulent mixing of components gives way to diffusive separation of gases. This altitude, at which the coefficients of molecular and turbulent diffusion are comparable, is called the turbopause [1,2,3]. Above the turbopause, ambipolar diffusion plays a decisive role in transport processes.

Turbulence and other atmospheric phenomena influence on the temperature of the lower thermosphere, which in turn affects the rate of chemical reactions in the atmosphere [4]. Studying turbulent phenomena in this region of the Earth’s atmosphere is a pressing task in the study of atmospheric chemistry, near-space dynamics, and radio wave propagation processes.

The turbopause level varies with the time of day, latitude, season, and solar activity [1,2,3,4,5]. Methods for measuring the turbopause height include radar measurements using mid-frequency radars ([3,6,7] and references therein), rocket experiments [3,7], and the use mid-, high- and very high-frequency radars [8]. In [2,6,9], mid-frequency radar to determine the turbopause was used. Scattered signals from ionization meteor trails were detected, and ambipolar diffusion coefficients were obtained from the radar echo decay time. Based on the application of various methods, it was established that the turbopause level varies mainly in the range from 80 to 120 km. In [9], the results of turbulent velocity measurements using the Buckland Park medium frequency (BPMF) radar (34.63° S, 138.48° E) at a frequency of 1.98 MHz. With the radar beam width, turbulent velocity varied primarily within the range of 1–4 m/s was presented. A method for studying atmospheric dynamics, temperature, and turbulence parameters using instruments installed on the SABER satellite offers significant potential [10].

In [11,12], Offermann et al. coined the term “wave turbopause,” defining it as a layer with lower and upper boundaries where maximum dissipation of atmospheric waves occurs. This was based on an analysis of temperature measurements using the SABER (Space Dynamics Laboratory of Utah State University (SDL), North Logan, Utah, USA) and CRISTA instruments (CRISTA manufacture University of Wuppertal, Wuppertal, Germahy) on the seasonal and latitudinal variability of the wave turbopause. They found that wave turbopause heights vary from 85 to 105 km (mostly around 90–95 km). The concept of the wave turbopause layer was further developed in [13,14]. In [14], a new algorithm for determining the wave turbopause based on numerical differentiation using atmospheric temperature data was proposed based on SABER/TIMED data.

Much attention is paid to the study of the influence of geomagnetic activity on atmospheric turbulence. In [2,6], it is noted that high geomagnetic activity causes increased wind velocity in the lower thermosphere and, as a consequence, changes in the turbopause height. In [11], it is shown that geomagnetic disturbances can intensify or alter the “wave turbopause” (the height at which the amplitude of vertical waves sharply increases).

The development of new measurement methods, the emergence of new instruments, the important role of digital data recording methods, and the use of neural networks are stimulating turbulence research in the MLT region. In recent decades, active research has been conducted into atmospheric dynamics and turbulence using one of the “heating” methods, which utilizes ionospheric disturbances by powerful radio emissions from a heating facility to determine many parameters of the undisturbed environment [15]. The API technique is one such method. It is based on the creation of artificial periodic irregularities in ionospheric plasma. Its distinguishing feature is the ability to study the neutral atmosphere and measure a large number of parameters of the neutral and ionized components of the ionosphere [15,16].

In this article, we present new results of determining the most important atmospheric characteristics in the MLT region, such as turbulent velocity and turbopause level, using the API method. The remaining sections of this paper are organized as follows. Section 2 provides a brief description of the method for studying the mesosphere and lower thermosphere based on the API technique. Section 3 describes the methodology and principles of data processing. Section 4 presents the processing results and examples of altitude–temporal variations in turbulent velocity and turbopause level obtained using the API method. Results of the measurement of the velocity of regular vertical motions in the MLT region are also presented. Section 5 discusses the obtained results and the manifestation of atmospheric waves. Finally, Section 6 contains conclusions.

## 2. API Technique

### 2.1. Basic Equipment

For many years, the API techniques have been developed on the basis of the SURA mid-latitude heating facility (56.15° N, 46.11° E).

To disturb the ionosphere and API formation, we used powerful transmitters of the SURA facility that create an electromagnetic field that disturbs the ionospheric plasma. The facility includes three high-frequency transmitters and a phased 144-element antenna array. The facility’s transmitters can emit high-frequency radio waves of ordinary and extraordinary polarization. The installation modules can operate independently. The antenna array measures 300 × 300 m. Each transmitter can emit powerful radio waves in the frequency range from 4 to 9.5 MHz with a maximum output power of 250 kW.

To create APIs, radio waves with extraordinary polarization in the 4–6 MHz frequency range are typically radiated. The frequency of the powerful wave is always below the ionospheric critical frequency. Ionospheric conditions are monitored using a CADI ionosonde (CADI manufacturer Scientific Instrumentation Limited (SIL), Saskatoon, SK, Canada) located at a point 1 km from the heating facility.

Currently, the SURA facility is also used as a source of probing pulses for diagnostics of the ionosphere. Two transmitters emit a powerful radio wave in phase continuously for several seconds, while the third transmitter emits probe radio waves in pulsed mode. To receive API scattered signals, a partial reflection facility with a 120 × 120 m phased-array antenna and specially designed receiver-recording equipment are used. For more detailed information about equipment of the SURA facility and the API complex, we recommend referring to [15,16,17].

The experiments were carried out using the SURA heating facility to create and diagnose inhomogeneities. The transmitters of the facility emitted into zenith a powerful extraordinary radio wave at a frequency of 4.7 or 5.6 MHz. The effective radiated power was 150–180 MW. API scattered signals were received by the phased antenna array of the partial reflections setup and their quadrature components were recorded [15,16]. The height and time resolutions were typically 0.15 km or 1.4 km and 15 s, respectively.

APIs are formed in the field of a powerful standing wave that occurs when the ionosphere is disturbed by powerful high-frequency (HF) radio emission [15,16]. Experimental and theoretical studies of the ionosphere by the API technique have shown that its application makes it possible to determine more than a dozen parameters of the ionosphere and the neutral atmosphere and to obtain a large amount of data on the dynamics and parameters of the ionized and neutral components of the Earth’s atmosphere in the altitude range from 50 to 60 km to the height of the F-layer maximum. Methods have been developed for determining many parameters of the ionosphere and the neutral atmosphere at these heights, including electron density, neutral temperature and some aeronomical parameters of the D-region and the sporadic E-layer (E_s_) [15]. In [16], a brief summary of recent studies of the Earth’s ionosphere at the heights of the mesosphere and lower thermosphere using the API method is presented.

### 2.2. API Formation and Diagnostics

APIs are formed as a result of uneven plasma heating in the field of a standing radio wave when a powerful wave is reflected from the ionosphere. The main features of API formation are described in detail in [15]. Note that, in different regions of the ionosphere, the formation of inhomogeneities is due to different physical processes. In the D-region (height 60–90 km), the temperature dependence of the coefficient of attachment of electrons to neutral molecules plays a key role in API formation. In the E-region (height 90–150 km), APIs are formed due to the diffusion redistribution of plasma under the action of an excess pressure of electron gas, and in the F-region (150–350 km), they are formed under the action of a ponderomotive force on the ionospheric plasma. Irregularities scatter probe radio waves. When the Bragg scattering condition is satisfied, the relatively high intensity of the backscattered signal is due to the in-phase summation of waves from each inhomogeneity [15]. Scattering of probe radio waves by APIs has resonant properties, that is, if the frequencies and polarizations of high-power and probe radio waves are equal, the amplitude of the signal scattered by inhomogeneities is 10–100 times higher than the level of natural noise. Therefore, the method was called the method of resonant scattering of probe radio waves on APIs. Many experiments using the SURA facility were carried out in order to determine the parameters of the ionosphere and the neutral atmosphere from the measured characteristics of API scattered signals. In particular, these parameters are the turbulent and regular vertical velocities and the turbopause level. Note that despite the fact that the values and direction of the vertical velocity often change, we call it a regular velocity as opposed to a turbulent (chaotic) one. For this purpose, the height profile of the relaxation time and phase of the signal scattered by inhomogeneities is used.

**Figure 1:**
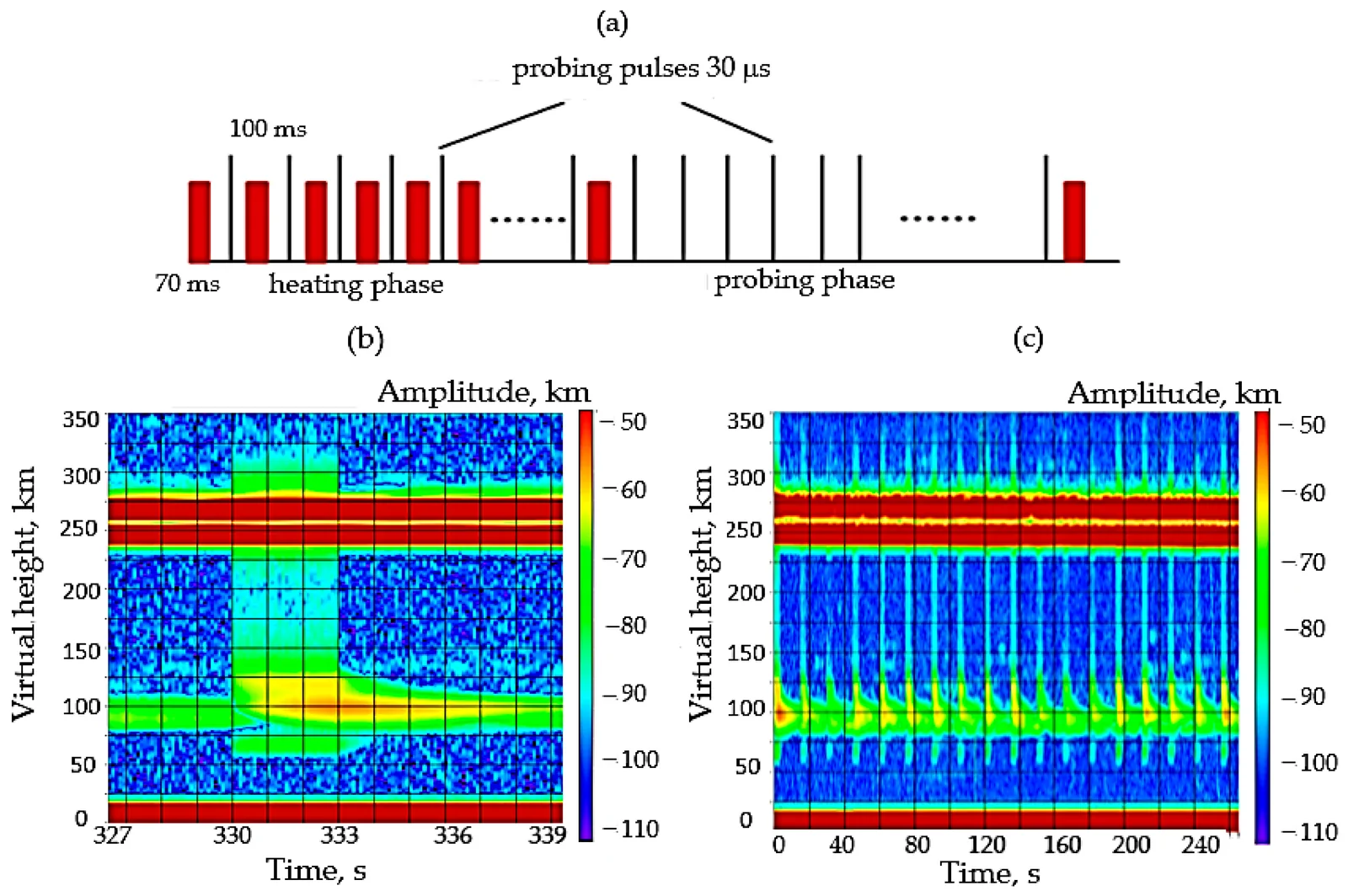
Diagram of the radiation of powerful and test radio waves by the SURA facility (**a**)—(taken from [16]) and an example of the API scattered signal on 7 October 2025 at the stages of development and relaxation of inhomogeneities for one session lasting 15 s (**b**) and a sequence of such sessions starting at 17:45:50 (**c**).

Figure 1a shows a diagram of the radiation of powerful and test radio waves by the SURA facility. Using different time schemes for the emission of high-power and probe radio waves, it is possible to study the API rise and decay (relaxation) processes [15,16,18,19,20,21,22]. Figure 1b,c show an example of the API scattered signal on 7 October 2025 at the stage of development and relaxation of inhomogeneities (and scattered signal) in coordinates of virtual height–time–amplitude.

During the heating time of 3 s, one can see the development of the API signal scattered in the altitude range of 60–270 km below the height of the specular reflection of the test radio wave. At a height of 60–75 km API signals from the D-region are seen; at heights of 80–160 km the signals scattered by APIs are seen in the E-region and interlayer E-F valley. The horizontal stripes at the top of Figure 1 are specular reflections of the probe radio wave of the ordinary and extraordinary polarization. Figure 1 shows the growth and decrease in the amplitude of the scattered signal during the development and destruction of inhomogeneities. The relaxation or decay time is determined when the amplitude of the scattered signal decreases by *e* times. In this example, the rise and relaxation times in the E-region reached 1.0–1.5 s. These values of API time scales are typical for the E-region, when ambipolar diffusion dominates in their formation and relaxation [15]. The API echoes of the F-region develop and disappear typically in less than 30–300 ms after the heater switching. Sometimes, after the end of the impact on the ionosphere, the relaxation of the scattered signal in the E-layer lasted for about 2–3 s.

After the end of the heating session of the ionospheric plasma by the powerful radio emission of the SURA facility in the E-region of the ionosphere (90–130 km), the APIs relax under ambipolar diffusion [15]. In this event, at the height of 90–130, the relaxation time τ is given by Formula (1)

(1) $$\tau =\frac{1}{K^{2}D}=\frac{M_{i}\nu_{im}}{k_{B}(T_{e}+T_{i})K^{2}}=\frac{M_{i}\nu_{im}}{2k_{B}{TK}^{2}},$$

where *D* is the coefficient of ambipolar diffusion, *k_B_* is the Boltzmann constant, *K* = 4π*n*/*λ* is the wavenumber of a standing wave in plasma, *λ* is the length of the emitted powerful radio wave and *n* is the refractive index, *M_i_* and *ν_im_* are the average mass of ions and the frequency of collisions of ions with molecules, and the temperatures of *T_i_* ions and *T_e_* electrons are equal to the neutral temperature *T* at the heights of the lower thermosphere.

In the D-region (60–90 km), the amplitude and relaxation time change in accordance with the temperature dependence of the electron detachment coefficient [15].

## 3. Methodology and Data Processing

Some results of determination of the turbulence parameters under various ionospheric conditions are presented in [15,20,21,22]. Here we present the results of determining the turbulent velocity and turbopause level under different natural conditions.

### 3.1. The Turbulence Impact on the Scattered Signal and Methodology

In the lower ionosphere, turbulence plays a significant role in atmospheric processes. Although it has been theoretically demonstrated that at altitudes of 90–130 km, relaxation of periodic irregularities occurs under the impact of ambipolar diffusion, experiments studying the ionosphere using the API method revealed that the altitude dependence of relaxation time does not always correspond to the diffusion law at all altitudes. It was established that the altitude diffusion dependence of the relaxation time is disrupted in the turbulent region [15].

**Figure 2:**
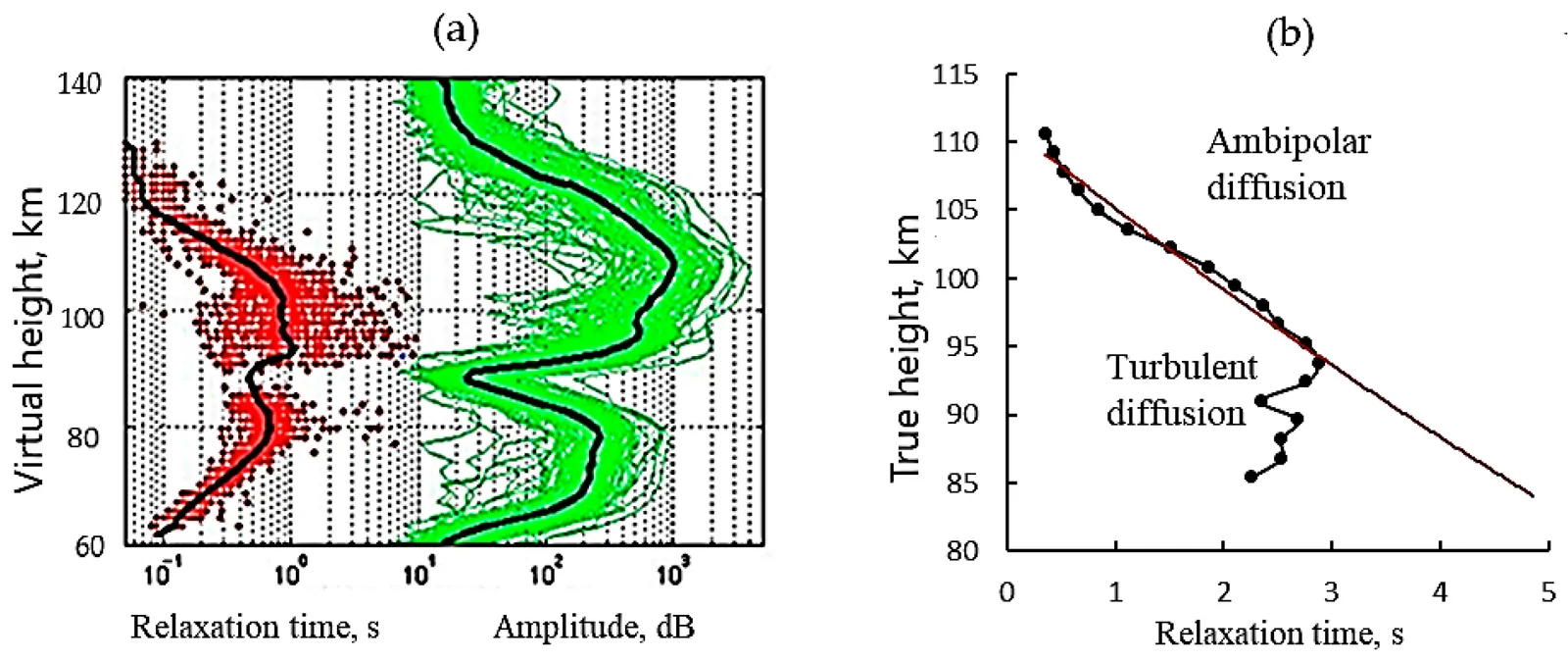
Experimentally measured dependences of the relaxation time and amplitude of the scattered signal in the session 18:50–19:00 LT on 9 August 2017 (**a**). The black lines correspond to the averaged profiles for this time interval. The typical height profile of the scattered signal relaxation time with the influence of turbulence below an altitude of 94 km (**b**). The red line shows the dependence of the relaxation time on height under the influence of ambipolar diffusion.

Turbulent motions affect the characteristics of the scattered signal, since they disrupt the ordered structure of the inhomogeneities, which causes dephasing of signals scattered by different parts of the scattering volume. As a result, the amplitude of the received signal decreases, and the relaxation process of the inhomogeneities occurs faster than under the influence of ambipolar diffusion. Figure 2 illustrates the effect of atmospheric turbulence on the relaxation time of the API scattered signal.

Note that, in Figure 2a, the dependence of the scattered signal amplitude and relaxation time is given as a function of the virtual height, as it occurs during measurement. In Figure 2b, the true height is plotted along the vertical axis. The dependence of the relaxation time on true altitude is used in the subsequent determination of turbulent velocity and the turbopause level. Figure 2a shows the experimentally measured dependences of the scattered signal amplitude and relaxation time for 9 August 2017. As follows from Figure 2, the relaxation time initially decreases smoothly with decreasing altitude and corresponds to the diffusion process. Figure 2b shows a typical height dependence of the API relaxation time, which is determined by the decrease in the scattered signal by *e* times. Below a certain altitude, this correlation is broken, and the relaxation time decreases more rapidly, due to the influence of turbulent diffusion. This altitude can be taken as the turbopause altitude *h_t_*. In this example, turbulence begins to affect the API below a true height of 94 km. Relaxation times below the turbopause altitude become significantly less than diffusion values. The sporadic E-layer increases the relaxation time due to an increase in the masses of ions, which are often positive metal ions, for example, Fe^+^ (56 aem) and Ca^+^ (40 aem) [23,24,25]. That is the local maximum of the amplitude and relaxation time of the scattered signal at a height of 95 km is due to the sporadic E-layer [15,16,19]. This can be clearly seen in Figure 2.

### 3.2. Data Processing

The method for determining the turbulent velocity is described in detail in [15,26]. The problem of the influence of atmospheric turbulence on the amplitude and relaxation time of the API under conditions of “frozen-in” turbulence was solved analytically. In addition, it was assumed that distortions of the periodic structure are created only by the field of the vertical component of the turbulent velocity (in the case of anisotropic turbulence) and the scattering volume is much larger than the spatial period of the inhomogeneities. In [26], an expression was obtained for the amplitude of the signal scattered by the entire volume of inhomogeneities, which turned out to be dependent on the distribution density of the turbulent velocity *V_t_*. The analysis was performed for three turbulent velocity distribution laws: normal, Cauchy, and hyperbolic secant. It is shown that the amplitude of the scattered signal depends in a complex way on the ratio of the times of turbulent and ambipolar diffusion. The simplest expression for the signal amplitude is obtained for the Cauchy distribution.

(2) $$A\left(t\right)=A_{0}{\left(1+\tau_{d}/\tau_{t}\right)}^{-1}\cdot exp\left[-\left(1/\tau_{d}+\sqrt{2}/\tau_{t}\right)t\right],\;$$

where $\tau_{d}(h)$—ambipolar diffusion time, $\tau_{t}(h)$—time due to turbulent diffusion. With such an expression for the amplitude of the scattered signal, its relaxation time *τ*, determined by the decrease in the measured signal amplitude by *e* times, is described by the formula $\tau^{-1}=\left(\tau_{d}^{-1}+\sqrt{2}\tau_{t}^{-1}\right)$, and the turbulent velocity is given by the formula

(3) $$V_{t}=({K\tau_{t})}^{-1}=\left(\tau^{-1}-\tau_{d}^{-1}\right)/K\sqrt{2}$$

According to Formula (3), the turbulent velocity was determined from the experimental data. To do this, it was necessary to know the diffusion relaxation time. It was determined by extrapolating the diffusion dependence $\tau_{d}(h)$ to heights below the turbopause level *h_t_.* Next, the value of $\tau_{d}$ was determined at each height and the turbulent velocity *V_t_* using Formula (3) [26].

To eliminate the error in determining the turbulent velocity due to the extrapolation of the altitude profile of the relaxation time to altitudes below the turbopause level, the experiment uses a method of creating APIs with different spatial scales alternately at two frequencies [15,16]. Typically, fixed frequencies are used, as a rule *f*_1_ = 4.75 and *f*_2_ = 5.6 MHz. Irregularities are created alternately at two frequencies *f*_1_ and *f*_2_, and the relaxation time profiles *τ*_1_ and *τ*_2_ of the scattered signal are determined for each frequency. Based on the ratio of the relaxation times for the two frequencies, the turbulent velocity and the ambipolar diffusion coefficient can be found independently of each other, eliminating the need to extrapolate the *τ_d_*(*h*) dependence [21,22].

## 4. Results

In this section we present results of experiments on the study of the turbulence of the lower thermosphere in different years.

### 4.1. Data Set

The data set for analysis includes the dependence of the scattered signal amplitude and relaxation time on true altitude. Each profile is obtained over a 15 s measurement session. Since the scattered signal always fluctuates significantly due to rapid variations in ionospheric parameters, we typically average the profiles over several minutes. Profiles that vary under the influence of the sporadic E-layer are excluded from the analysis. We use the following data processing procedure. First, the scattered signal relaxation time is calculated at each altitude based on the amplitude reduction by a factor of *e* times. Then, the altitude profiles are averaged over 5–10 min to reduce random errors. Virtual altitudes are converted to true altitudes, and the relaxation time versus true altitude is plotted. The linear relationship that determines *τ*(*h*) above the turbopause level is extrapolated to lower altitudes. The turbulent velocity *V_t_* is determined using Formula (2). At the turbopause altitude, it becomes zero.

Measuring the phase of a signal scattered by artificial periodic irregularities allows one to directly measure the vertical plasma velocity. However, in the lower ionosphere, the plasma is a minor admixture and moves along with the neutral component. Therefore, by measuring the vertical plasma velocity *V* we measure the vertical velocity of the neutral environment.

The vertical plasma velocity *V* is determined by measuring the phase *φ* of the scattered signal as

(4) $$V=\frac{\lambda }{4\pi }\frac{d\varphi }{dt}=\frac{c}{4\pi fn}\frac{d\varphi }{dt},$$

where *f* is the frequency of high-power and probe radio waves, *n* is the refractive index and *c* is the light velocity in vacuum. A negative velocity value means upward movement.

The results of experimental data processing are presented in Table 1. Table 1 shows the date and interval of observations, the range of the turbopause *h_t_* variations and the turbulent velocity *V_t_* values averaged over a 5 min interval. The next column shows the average values of the regular vertical motion velocity *V* at the turbopause altitude and the average values <*V*> for each day of observations in the altitude range of 85–130 km. A negative value of the vertical velocity corresponds to upward motion. The geomagnetic index K_p_ and the sunspots number W during the period of the experiment are also given.

Most of the data presented in Table 1 were obtained in the autumn months of 2016–2019 during daytime hours, as well as sunset and sunrise hours, on 12–13 August 2015. The geomagnetic field during these measurements was generally calm or slightly disturbed. The geomagnetic coordinates of the SURA facility varied in the range of (50.715–50.85)° N, (129.476–129.31)° E for the events listed in Table 1. The average solar activity level was recorded during the sunset-sunrise hours of 12–13 August 2015.

### 4.2. Turbulent Velocity

**Figure 3:**
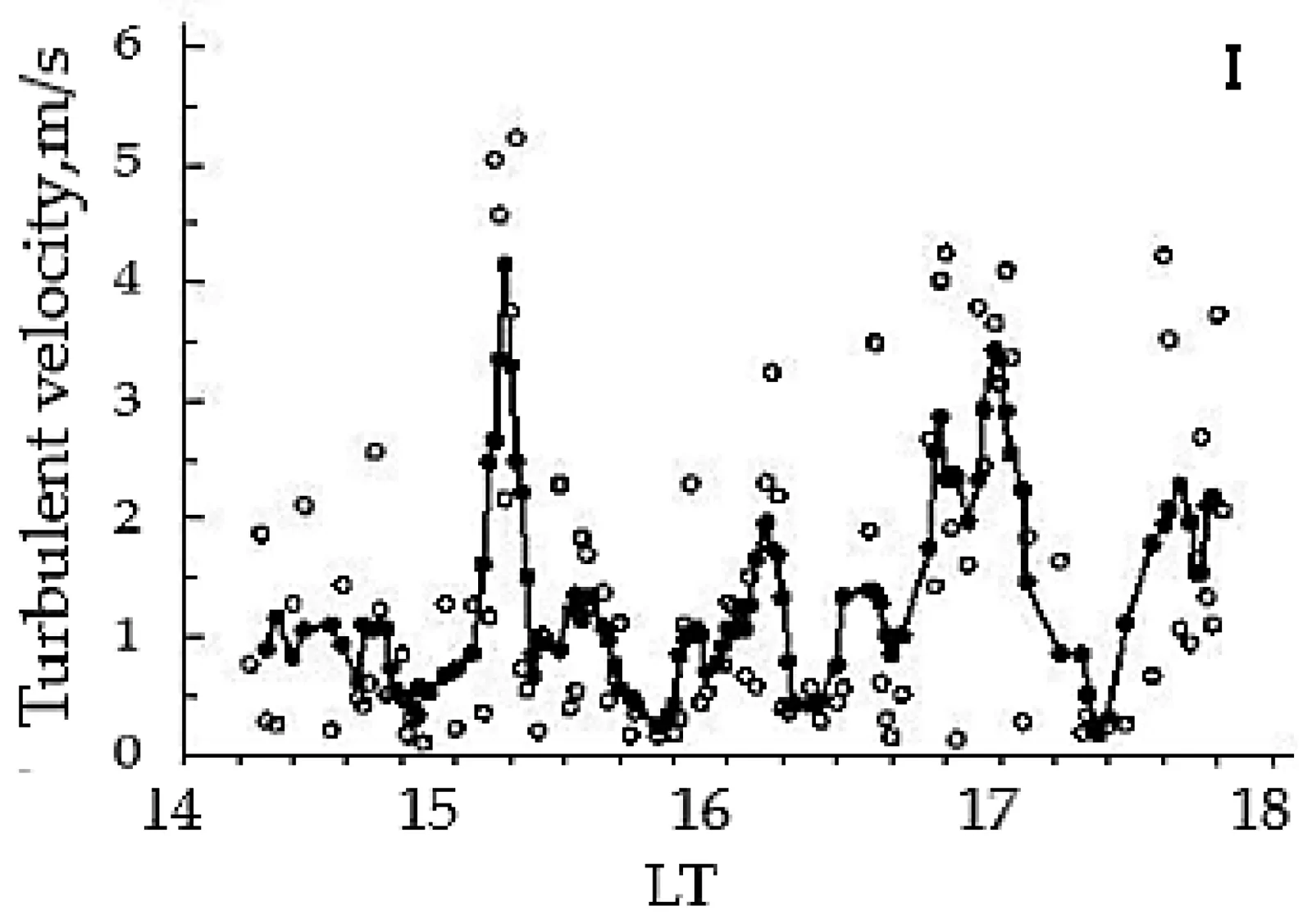
Dependence of turbulent velocity *V_t_* on time at the altitude of 100 km on 12 August 1999. The circles correspond to signals recorded every 15 s, and the curve with points corresponds to data smoothed by the moving average method over a 5 min interval. The vertical segment shows the measurement error. The figure is taken from [15] and modified.

The results of the first trial measurements of turbulent velocity are presented in [15]. They showed that the developed method yields reasonable velocity values. Furthermore, rapid variations in velocity were detected, requiring a data averaging procedure. The results of the trial experiment are shown in Figure 3. In subsequent years, the receiving equipment was upgraded, and the processing of API experiment data was optimized. Figure 3 shows time dependences of turbulent velocity *V_t_* obtained at an altitude of 100 km on 12 August 1999.

The purpose of averaging is to smooth out the smallest velocity fluctuations. Figure 3 shows fluctuation ranges of the turbulent velocity from 0.5 to 5.0 m/s with wave-like periods ranging from 10 to 90 min. It should be noted that such large variations in the turbulent velocity could be due to natural non-stationary processes rather than measurement errors, which amounted to no more than a few cm/s [15]. Furthermore, in [15] it was concluded that turbulence is intermittent based on the high correlation between turbulent velocity and regular vertical velocity at higher altitudes. Intermittency may be caused by the “throw” of inhomogeneous regions with developed turbulence to higher altitudes through vertical transport. Measurements showed large variations in turbulent velocity in the scattering volume, which may indicate a non-stationary process. In the smoothed curve, the wave-like variations in turbulent velocity over time are also clearly visible.

**Figure 4:**
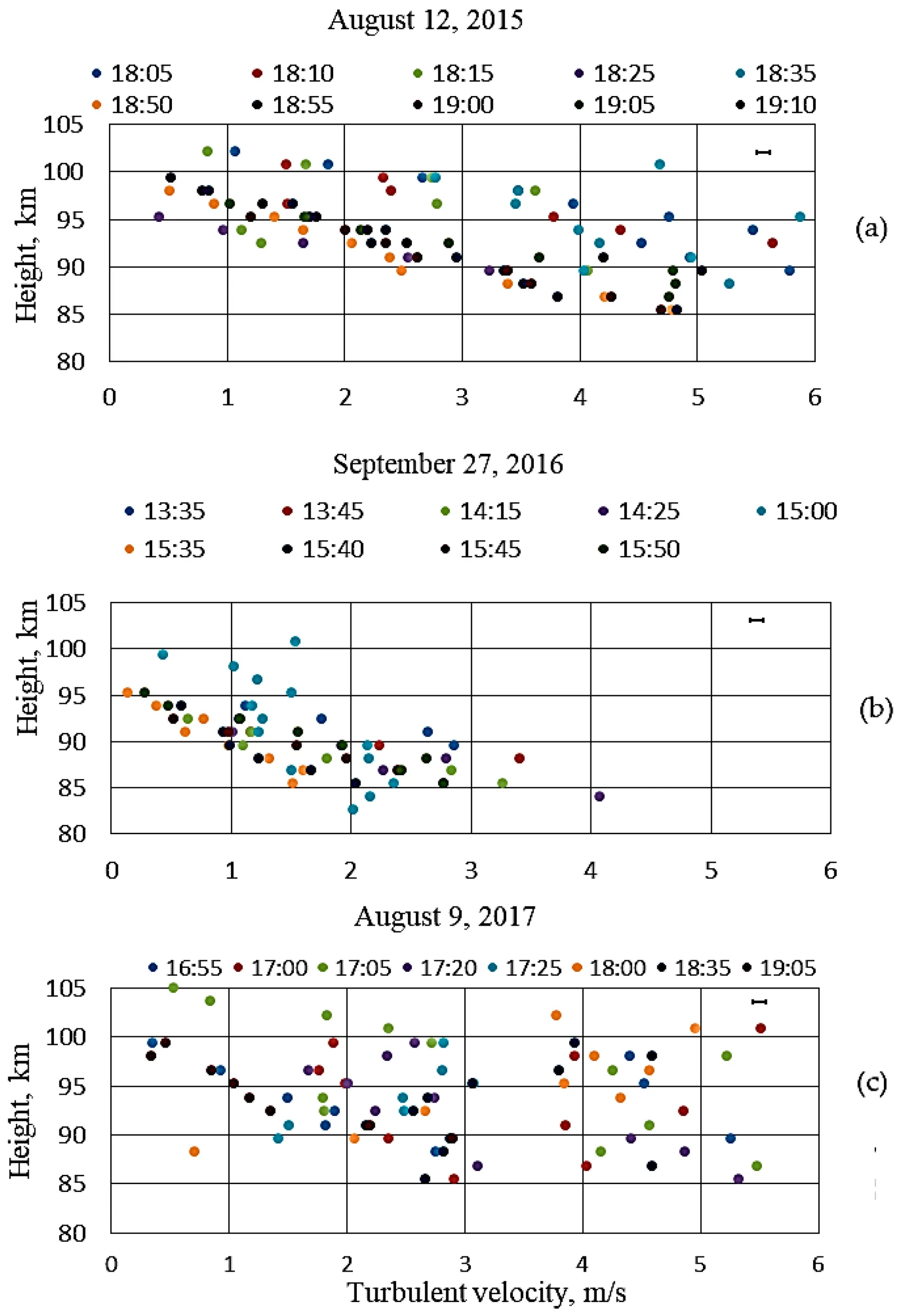
Turbulent velocity versus height below the turbopause on 12 August 2015 (**a**), 27 September 2016 (**b**) and 9 August 2017 (**c**). Dots of different colors correspond to different measurement sessions. The horizontal segment shows the measurement error of the turbulent velocity.

**Figure 5:**
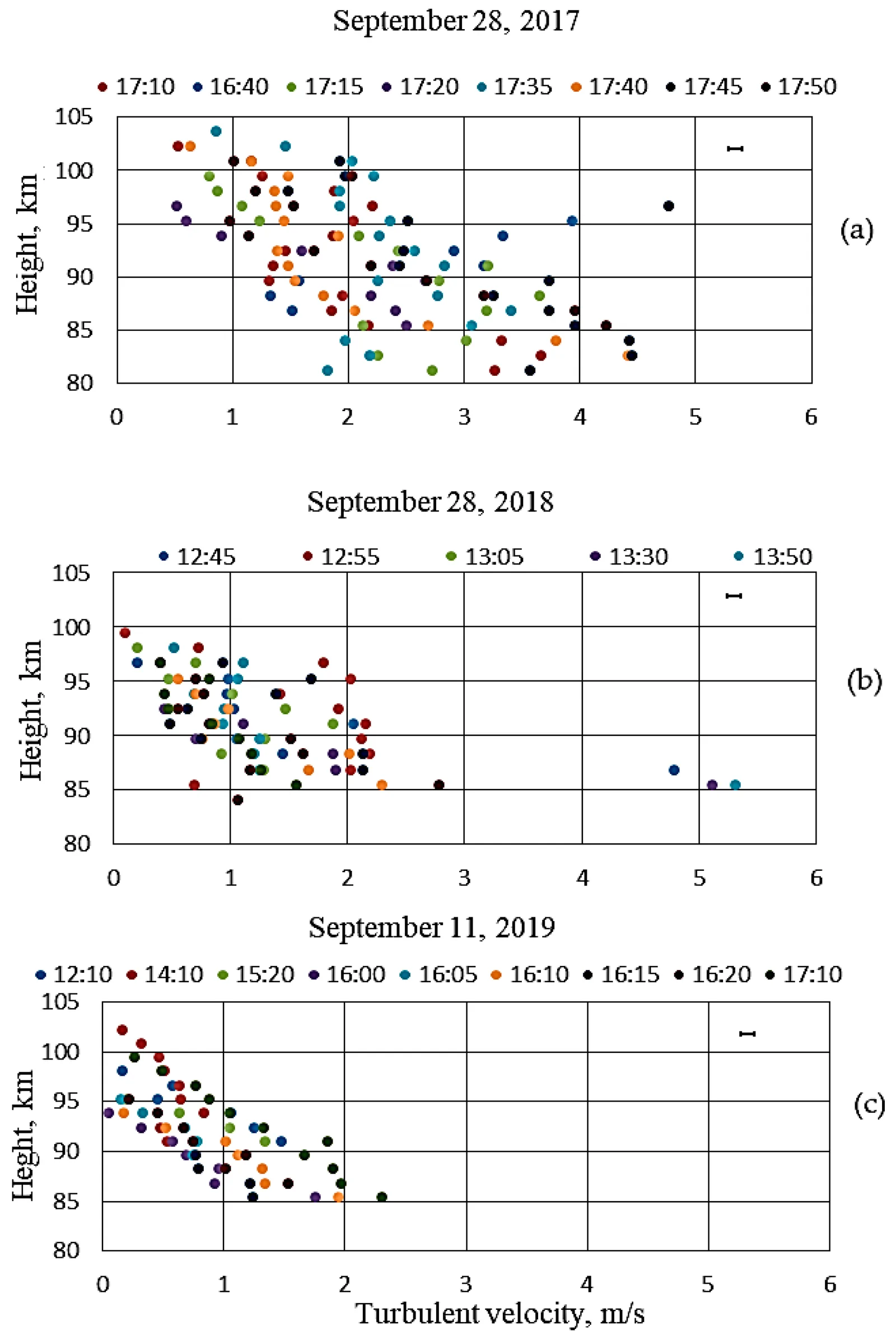
Turbulent velocity versus height below the turbopause level on 28 September 2017 (**a**), 28 September 2018 (**b**) and 11 September 2019 (**c**). Dots of different colors correspond to different measurement sessions. The horizontal segment shows the measurement error of the turbulent velocity.

Figure 4 and Figure 5 show turbulent velocity variations obtained in different years of observations using the API method, according to the algorithm described above. Each point on the graph is obtained by averaging the scattered signal relaxation time over a 5 min interval. The date and start of the measurement session are indicated on each graph.

Figure 4 and Figure 5 show that turbulent velocity varied primarily from 0 to 2–3 m/s, increasing to 5–7 m/s at certain points in time. Some key features of turbulent velocity changes can be identified. We note high variability in turbulent velocity within a single measurement session and over time. Turbulent velocity reaches its minimum value at the turbopause altitude. As altitude decreases into the region of developed turbulence, turbulent velocity increases. Extremely high velocity values of up to 5–7 m/s were occasionally observed, primarily at altitudes of 82–85 km. In these cases, turbulent velocity was comparable in magnitude to the velocity of the plasma regular vertical motion (see Section 4.3). Occasionally, such values of the velocity were also observed at altitudes of 90–100 km. The cause of this is still unclear. Strong variability in turbulent velocity at the same altitude is evident, related to atmospheric wave dynamics.

### 4.3. Turbopause Level

As follows from Table 1, the turbopause level *h_t_* varied in the altitude range from 88 to 108 km with an average value of 100–102 km. The minimum turbopause level sometimes dropped to a height close to the mesopause level. No significant dependence on time of day was found.

**Figure 6:**
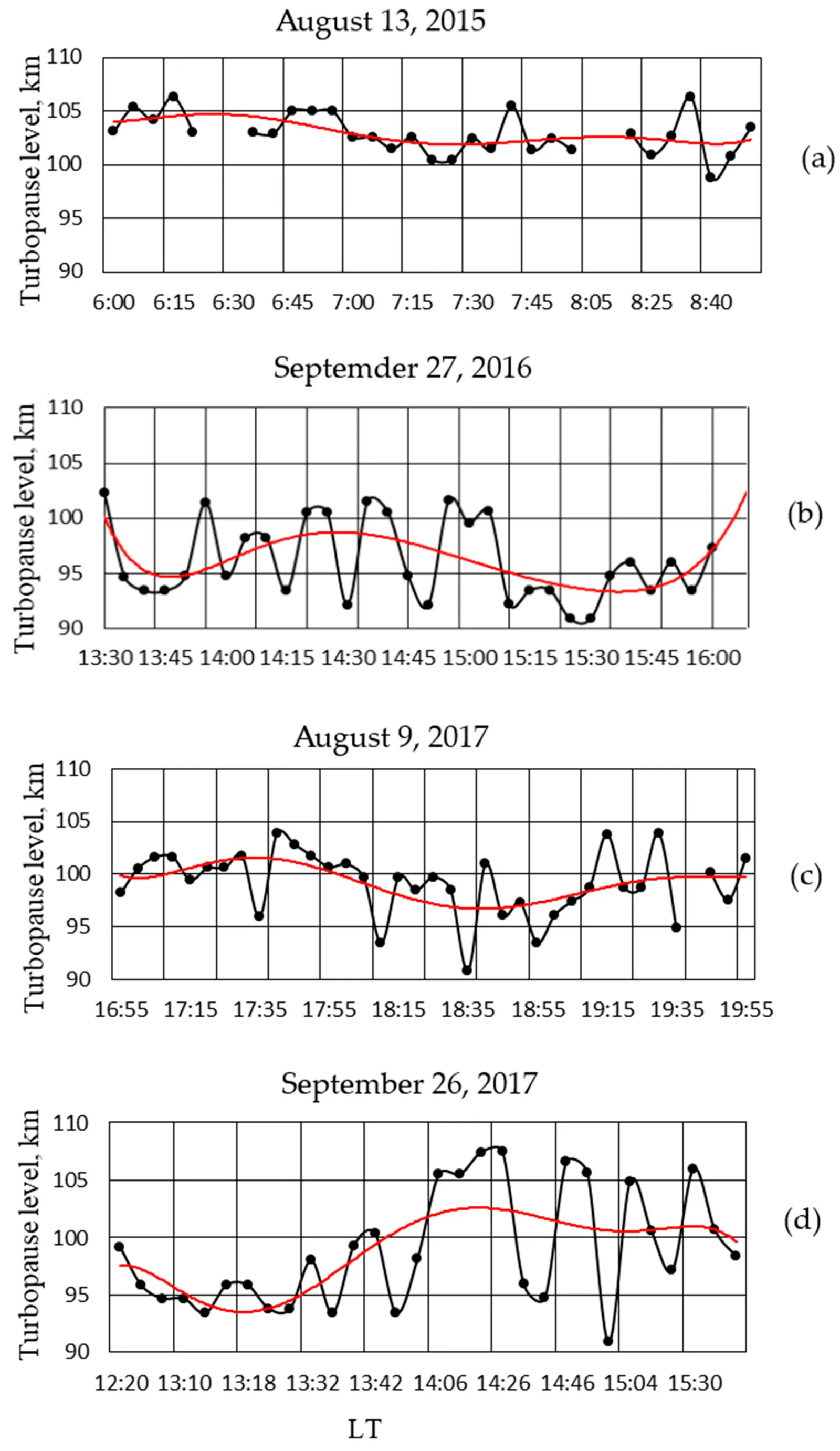
Variations in the turbopause level on 13 August 2015 (**a**), 27 September 2016 (**b**), 9 August 2017 (**c**) and 26 September 2017 (**d**) and its wave-like oscillations. Each point in the figures is obtained by averaging data over 5 min intervals. The red lines show the sixth-order polynomial trend. The variability of the turbopause level over time and the wave-like nature of the changes are clearly visible.

**Figure 7:**
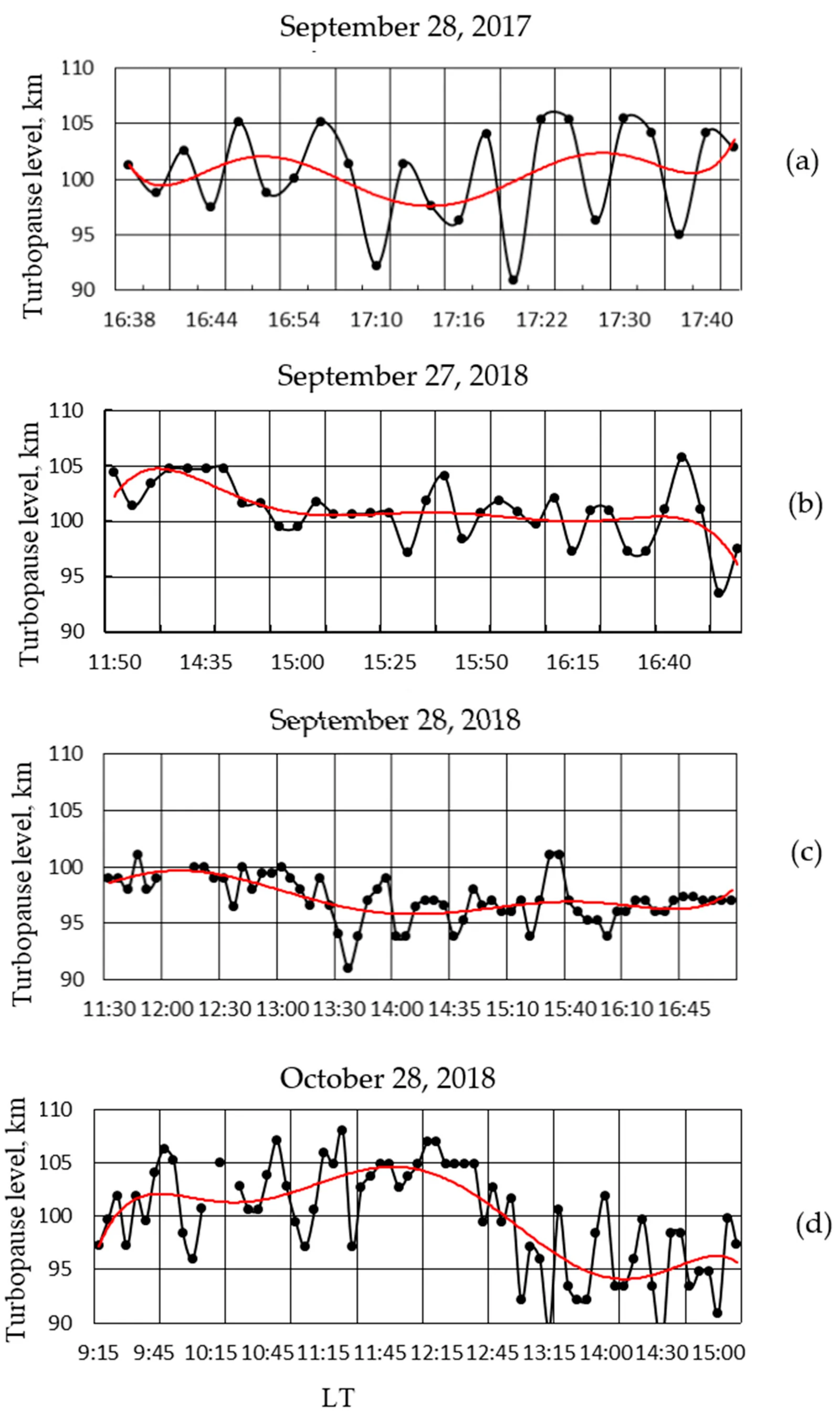
Variations in the turbopause level on 28 September 2017 (**a**), 27 September 2018 (**b**), and 28 September 2018 (**c**) and 28 October 2018 (**d**) and its wave-like oscillations. Each point in the figures is obtained by averaging data over 5 min intervals. The red lines show the sixth-order polynomial trend. The variability of the turbopause level over time and the wave-like nature of the changes are clearly visible.

In Section 2, we demonstrated that the amplitude of the scattered signal under the influence of atmospheric turbulence begins to decrease at the turbopause level. Our first results on determining the turbopause level *h_t_* based on the results of ionospheric research using the API method in 2007–2014 can be found in [22]. It was shown that in these measurements, the turbopause level varied in the range of 94–106 km with an average level from 99 to 102 km and a decrease in the evening hours to 94 km. Wave-like variations were observed with periods from 10–15 to 30–40 min typical of internal gravity waves (IGWs). Figure 6 and Figure 7 show the temporal dependence of the turbopause level for several days.

Taking into account our previous measurements [20,22], it was found that the turbopause level was in the altitude range of 85–110 km, while the lower boundary of the turbopause in most measurements was at an altitude of 90–91 km. The minimum low level of the turbopause dropped almost to the altitudes of the mesopause.

Increasing fluctuations in the turbopause level were observed on 26 September 2017, with a maximum value of 107 km. During sunrise on 13 August 2015, the turbopause level varied from 99 km to 106 km, fluctuating around an altitude of 102 km.

Similar wave-like variations in the turbopause level in studies of the ionosphere and neutral atmosphere using the API method are presented in [20,22]. The influence of atmospheric waves is noted in virtually all known publications on experimental studies of atmospheric turbulence. In [22], information is provided indicating that an increase in the turbopause level to 110 km was observed with a significant increase in the temperature of the neutral component, also measured using the API method. It was concluded that this phenomenon could be caused by the development of convective instability.

A common feature of the turbopause temporal change is variability over time and wave-like variations. These variations were most pronounced on 27 September 2016, 26–27 September 2017, and 28 September 2018. On these days, both relatively rapid variations with periods of 5 to 15 min and longer ones with periods of 30 min to two hours or more were observed. It should be noted that such periods of change in the characteristics of the ionosphere and neutral atmosphere may be caused by the propagation of internal gravity waves [2,3,4,5,7,27].

In [2], based on observations over several years using meteor radar, data are presented on the height of the turbopause at 52° N, which varied over the course of a year from 95 km to 107 km, with individual values increasing to 110 km.

### 4.4. Regular Vertical Plasma Velocity

The API technique allows one to measure a large number of parameters of the ionosphere and neutral atmosphere in one experiment, including the vertical plasma velocity, which is equal to the velocity of the neutral medium at the heights of the mesosphere and lower thermosphere [15,16,19]. It should be noted that vertical motion of the medium is one of the components of atmospheric dynamics.

Many results of the API measurements of the vertical velocity in different years, under different ionospheric conditions and natural phenomena are presented in [15,16,19]. In [15], it is reported that the average monthly velocity values were about 1 m/s at altitudes below 100 km, increasing to 5 m/s with increasing altitude.

**Figure 8:**
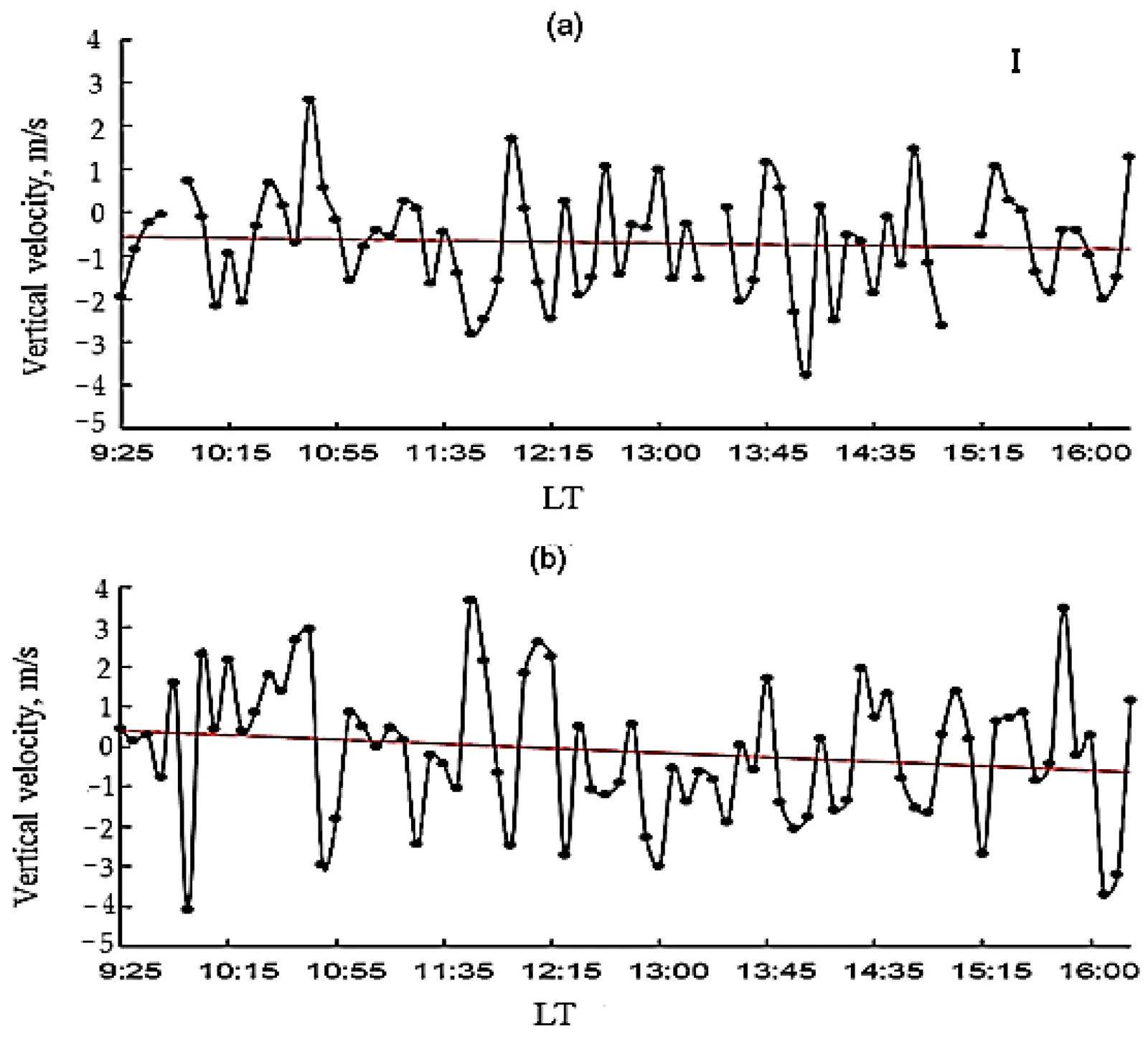
Temporal dependences of the velocity of the regular vertical plasma motion at the height of 100 km (**a**) and 105 km (**b**) on 26 October 2018. Each point corresponds to a 5 min data average. The red line shows a linear approximation of the data. The vertical segment shows the measurement error of the vertical velocity.

Below, we present examples of the measured regular vertical velocity. Figure 8 shows temporal variations in vertical velocity at altitudes of 100 km (a) and 105 km (b) on 26 October 2018. The red line shows a linear approximation (linear trend) of the data. The irregular change in velocity magnitude and direction with quasi-periodic variations is clearly visible.

In the example shown in Figure 8, the height of 100 km corresponds to the average turbopause height for that day, and the height of 105 km is outside the turbulent region. Regular vertical motions in the MLT region are characterized by rapid variations in the magnitude and direction of velocity during an individual measurement over 15 s. Velocity values at individual moments reached 10 m/s or more during a single measurement, and velocity values averaged over a five-minute interval typically varied, as a rule, from –5 m/s to +5 m/s (positive velocity values indicate downward motion). Our long-term experimental and theoretical studies have shown that such vertical velocity values are caused by wave motions in the atmosphere. As shown in [15], internal gravity waves (IGWs) can contribute to vertical velocity variations of up to 10–12 m/s. In the studies [15,16,18,19,21], wave motions with a period from 5–10 min to 4–5 h were found, and the period (scale) of the waves in height was 5–25 km. The periods of the waves contributing to the velocity variations correspond to IGWs [28,29,30,31,32,33].

**Figure 9:**
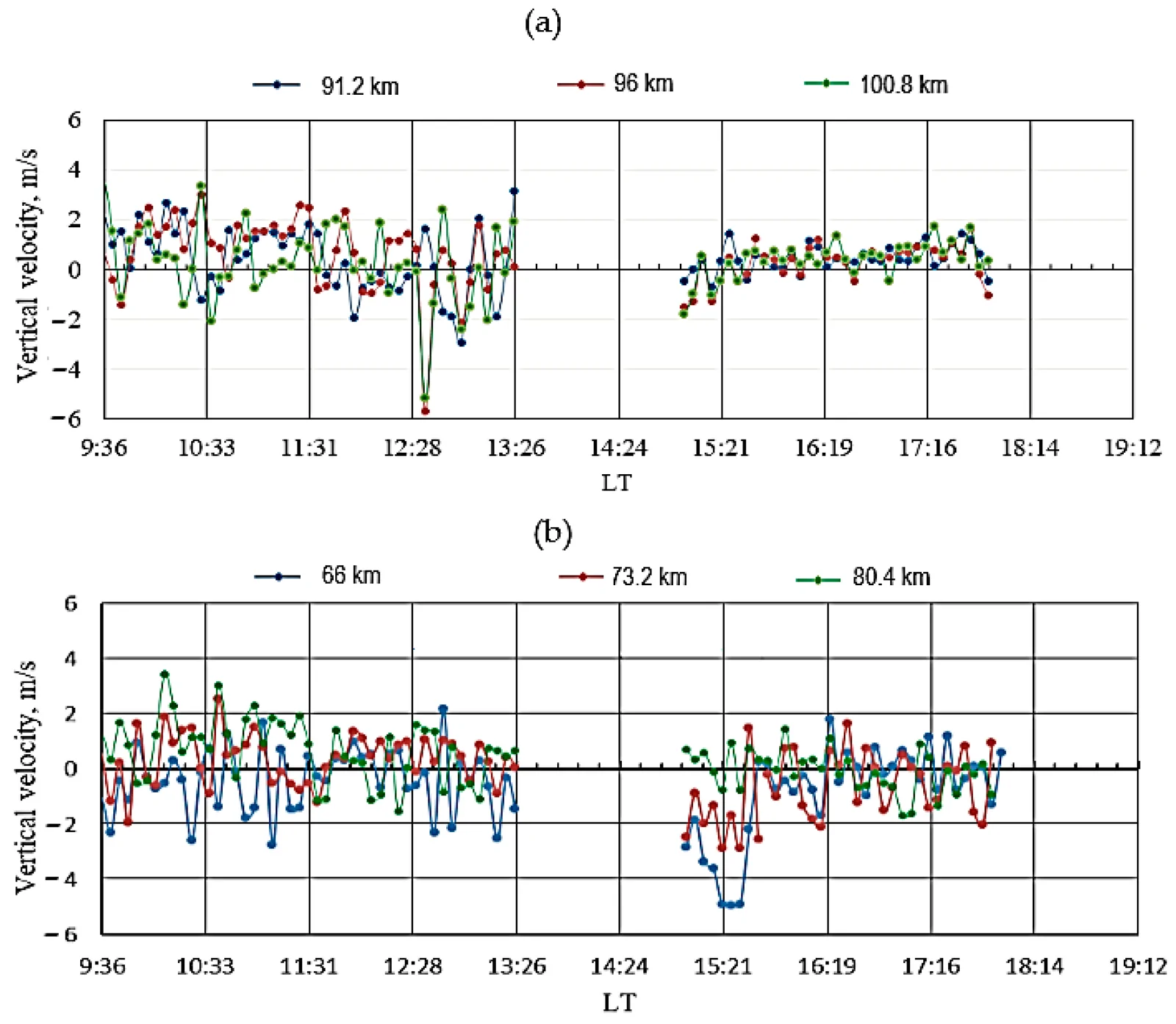
Temporal dependences of the velocity of the regular vertical plasma motion on 7 October 2025 in the E-region at altitudes of 91.2 km, 96 km and 100.8 km (**a**) and in the D-region at altitudes of 66 km, 73.2 km, 80.4 km (**b**); each velocity value was obtained by averaging over a 5 min time interval.

Figure 9 shows the results of recent measurements of vertical velocity at mesosphere (a) and lower thermosphere (b) altitudes. Rapid and profound variations in vertical velocity can be seen in Figure 9. At mesospheric altitudes of 66–88 km and at lower thermosphere altitudes of 91.2–100 km, the range of velocity variations decreased in the afternoon hours.

In some sessions, the scatter of velocity values in adjacent 5 min measurements reached 5–7 m/s, with averaging leveling out the more significant spread of instantaneous (in a single measurement) values. The period of wave-like variations typically increases with height, which is characteristic of IGWs [4,28,29].

As follows from Figure 8 and Figure 9, the vertical velocity almost always changes sign with height, passing through zero. When the direction changes with increasing height from ascending to descending movements, conditions are created for the redistribution of positive metallic ions in the Earth’s magnetic field in a non-uniform velocity field and the formation of a sporadic E-layer according to the wind shear [34,35,36,37]. The impact of turbulent irregularities on the semi-transparency of the sporadic E-layer (E_s_) is discussed in [38]. According to observational results, this height (the transition of the velocity through zero) corresponds, as a rule, to the height of the maximum of the sporadic E-layer. In addition, this height with a zero vertical velocity value is often close to the height of the turbopause sometimes.

From Table 1 and Figure 3, Figure 4, Figure 5, Figure 8 and Figure 9, it follows that the velocities of turbulent and regular vertical motion are comparable.

**Figure 10:**
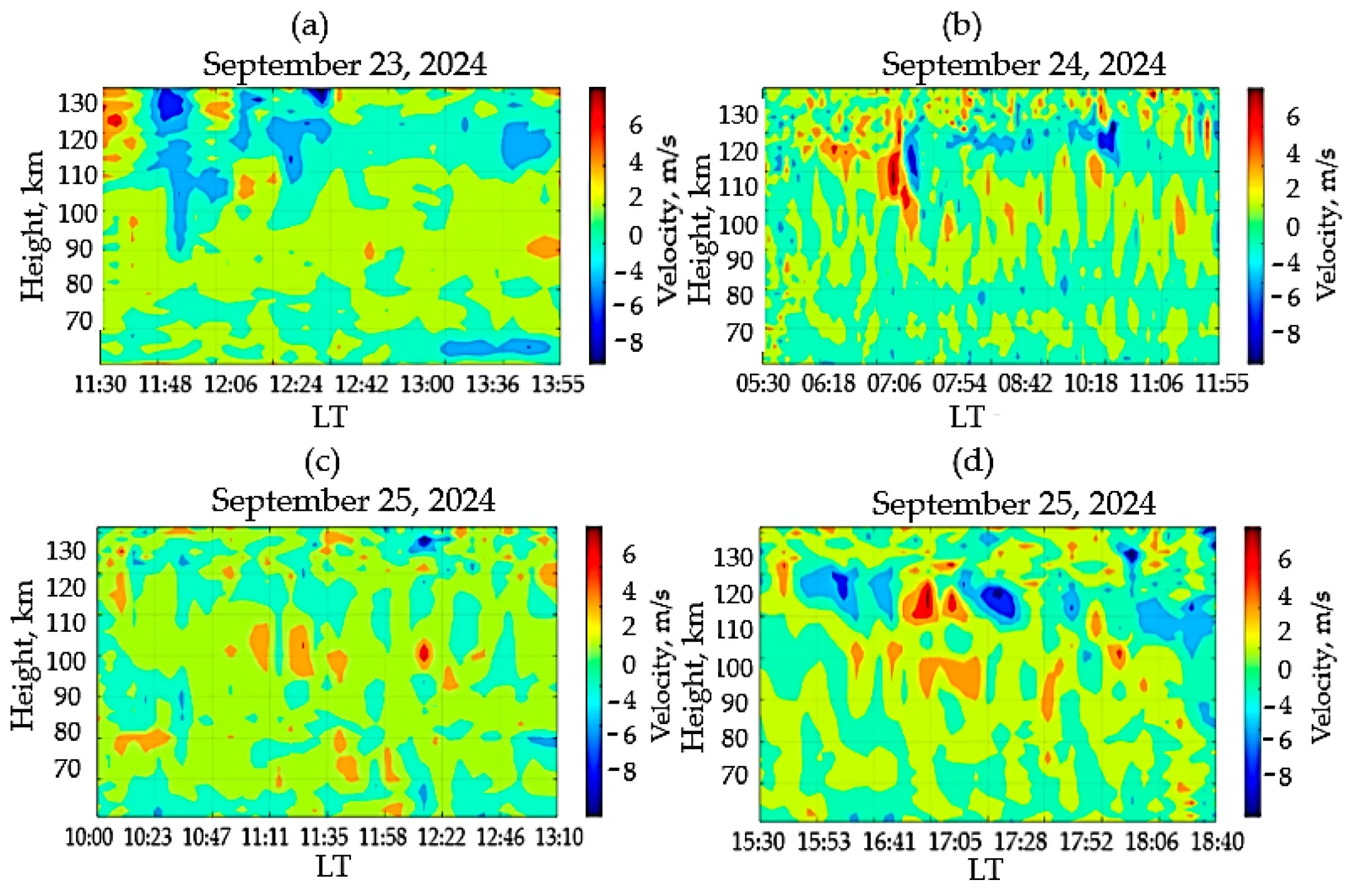
Altitude–temporal variations in the velocity of regular vertical motion on 24–25 September 2024, at altitudes from 60 to 125 km: (**a**) 23 September 2024 from 11:30 a.m. to 1:55 p.m., (**b**) 24 September 2024 from 6:30 a.m. to 11:55 a.m., 25 September 2024 (**c**,**d**) from 10:00 a.m. to 1:10 p.m and 3:30 p.m. to 6:40 p.m.

**Figure 11:**
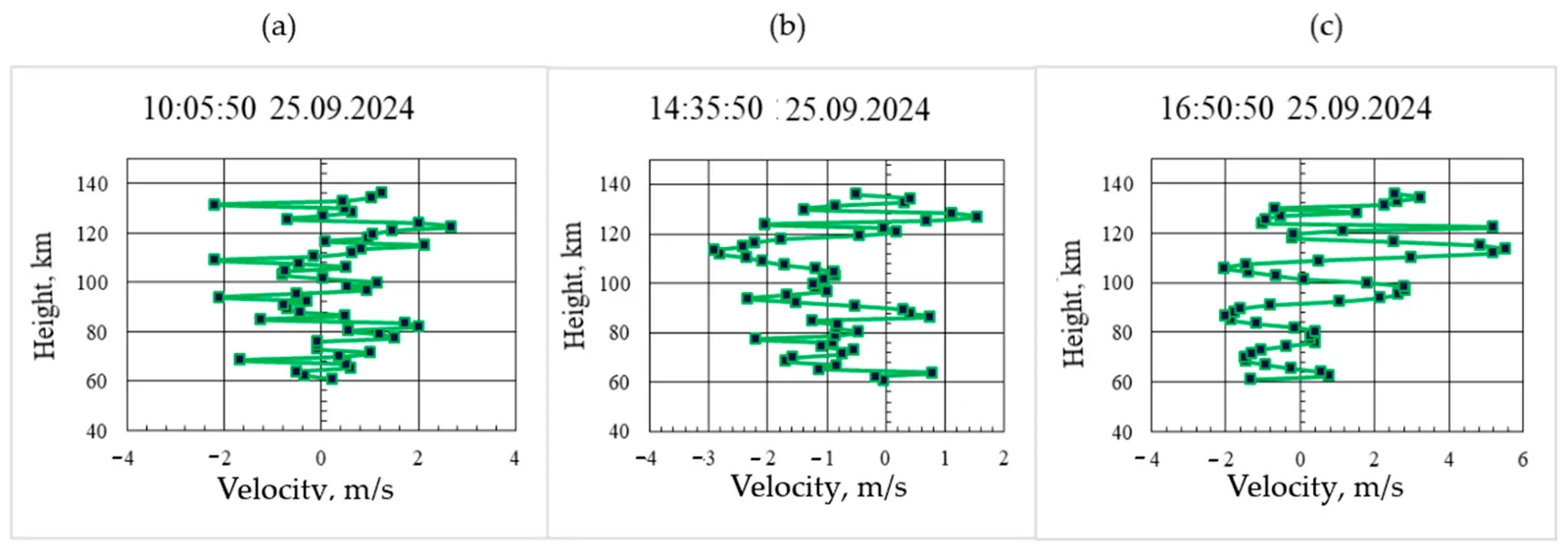
Examples of velocity variability with altitude at different times on 25 September 2024, with data averaged over two-minute intervals: (**a**) 10:05:50 a.m., (**b**) 2:35:50 p.m., (**c**) 4:50:50 p.m. Note that negative velocity values indicate upward motion.

**Figure 12:**
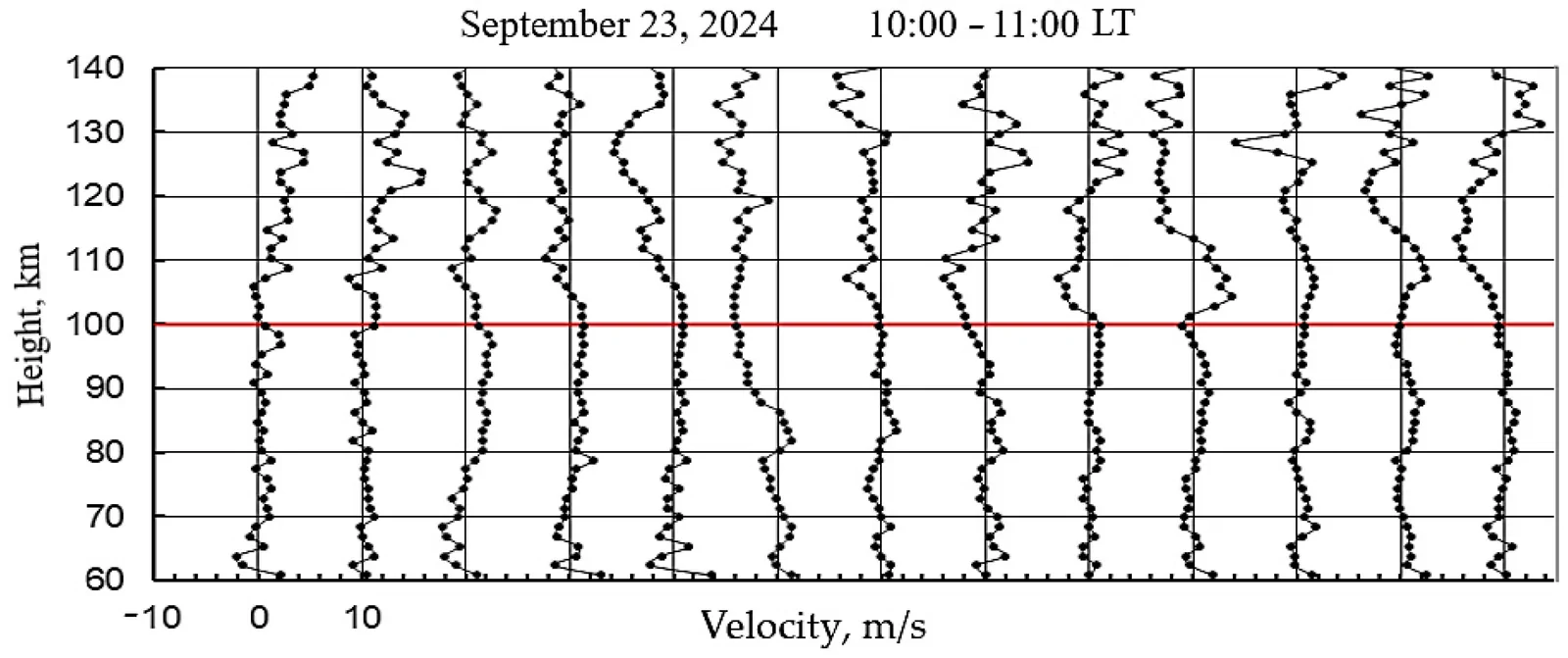
Vertical velocity altitude profiles averaged every 5 min for an hour on 23 September 2024. The red line marks the 100 km height.

Figure 10, Figure 11 and Figure 12 show the results of measurements of the regular vertical motion velocity in the MLT region, measured using the phase of the API scattered signal, in September 2024. In most cases, the data presented correspond to velocity values averaged over 5 min intervals. Figure 8 shows velocity variations at altitudes of 60–125 km in altitude–velocity–time coordinates. Figure 9 shows examples of velocity variability with altitude on different days. Figure 10 shows vertical velocity altitude profiles averaged over 5 min.

Measurements on 23 September 2024 (Figure 10a) cover the hours around midday, from 11:30 a.m. to 1:55 p.m. Below 110 km, downward motions predominated for most of the time (positive velocities). At certain points, vertical downward motions with a velocity of approximately 3 m/s were observed. Above 110 km, ascending and descending motions alternated. In the mesosphere below 90 km, motions with a velocity of approximately 1 m/s in both directions alternated.

A different picture was observed on 24 September 2024 (Figure 10b), in the morning hours from 6:30 a.m. to 11:55 a.m. Velocities varied from −6 m/s (upward motion) to +6 m/s (down motion), with constant changes in direction throughout the entire period. In the late afternoon from 10:00 a.m. to 1:10 p.m. and early evening hours from 3:30 p.m. to 6:40 p.m on 25 September 2024 (Figure 10c,d), the most intense changes in direction occurred in the altitude range from 110 to 120 km, with velocities ranging from −8 m/s (upward movement) to +6 m/s (downward movement).

Vertical velocity profiles measured at 150 m intervals (Figure 11) show a rapid change in velocity sign (during the measurement session), i.e., a change in direction. This signifies a reversal of the plasma’s vertical motion from ascending to descending and vice versa. This is a manifestation of ionospheric dynamics.

At mesospheric altitudes (60–90 km), the vertical plasma velocity changes with altitude and time. For example, in the session shown in Figure 11a, velocities were clustered around zero across the entire altitude range. In the session shown in Figure 11b, predominantly negative velocity values of up to −2.3 m/s were obtained, indicating upward motion. Furthermore, a wave-like change in velocity with altitude can be seen. This is how an atmospheric wave, such as an IGW, can propagate from the lower atmosphere to the ionosphere. Figure 11c demonstrates an increase in velocity with altitude and a change in the predominant direction of motion above 100 km.

Sporadic layers are typically observed at altitudes above 90 km [34,35,36,37]. In the example in Figure 11a,c, such conditions were observed at altitudes of 95–100 km. This, in turn, created conditions for the formation of a sporadic E-layer. Indeed, this layer was detected by both the ionosonde and the API method. The scattered signal from the E_s_ layer is shown in Figure 2b at an altitude of approximately 100 km.

Figure 12 shows vertical velocity profiles measured sequentially every 5 min during the hour from 10:00 to 11:00 LT. It is evident that the greatest changes in the magnitude and direction of the velocity mainly occurred above 100 km. Figure 10 clearly shows that the greatest variability in vertical velocity in this measurement session was observed above 100 km, most likely under the influence of atmospheric waves.

## 5. Discussion

We have convincingly demonstrated using the API method that atmospheric waves influence the characteristics of scattered signals and, consequently, the parameters of the ionosphere and neutral atmosphere, such as temperature, density, electron concentration, vertical and turbulent motion velocities, turbopause level, etc. [15,16].

**Figure 13:**
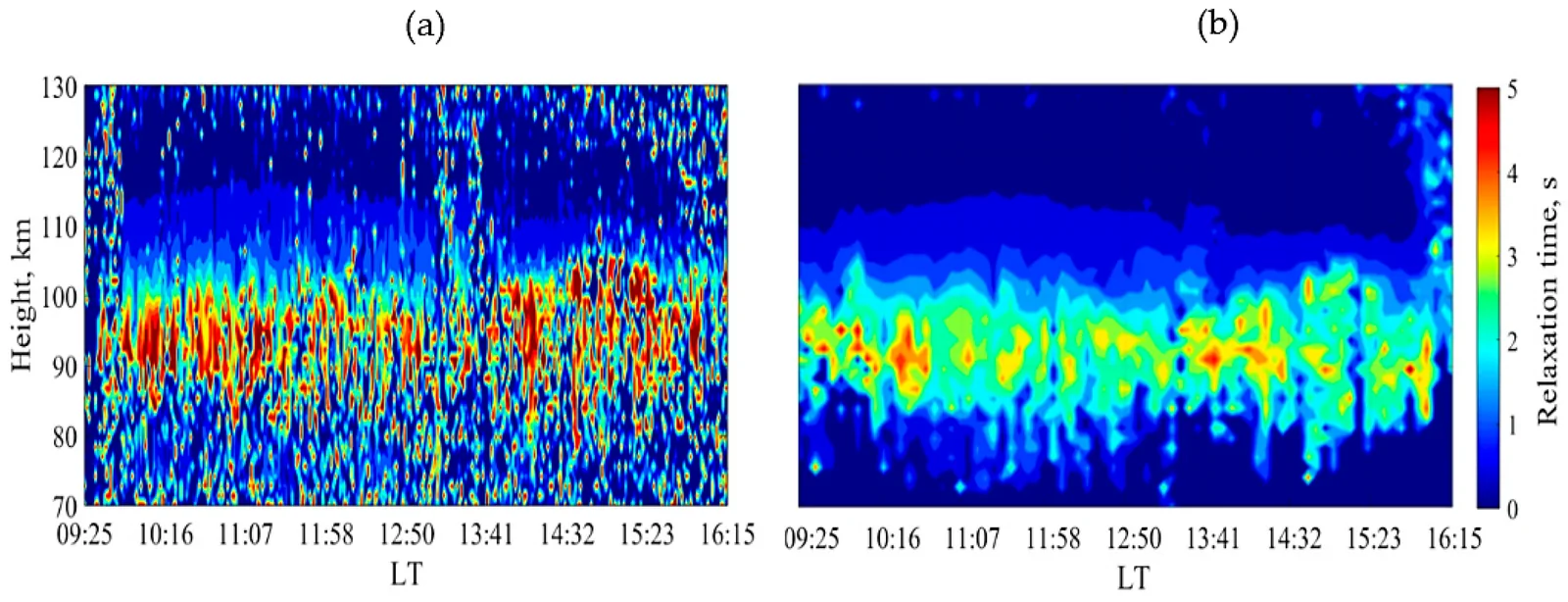
Temporal-height dependence of the relaxation time in the lower ionosphere on 25 October 2018. (**a**) Shows the relaxation time averaged over a 1 min interval. (**b**) Presents smoothed data over a 5 min interval. With increasing altitude, the relaxation time of the diffuse signal decreases in accordance with the diffusion law. Wave-like variations are caused by the propagation of atmospheric waves.

A typical example of atmospheric wave manifestations in temporal variations in the scattered signal relaxation time is shown in Figure 13. Figure 13a shows the relaxation time averaged over a 1 min interval. Figure 13b presents smoothed data over a 5 min interval. Wave oscillations lasting from 15 min to an hour or more are clearly visible. The periodic increase in relaxation time to 4.0–4.5 s at an altitude of approximately 95 km is due to the influence of a translucent sporadic E-layer (E_s_), which appeared at that time over the observation site and was recorded by the ionospheric ionosonde CADI. The values of the relaxation time of the scattered signal at different altitudes correspond to the theory of the API formation in the lower ionosphere [15]. Oscillations with periods greater than 5 min are usually associated with the propagation of IGWs [28,29,30,31].

Comparing Figure 13a,b demonstrates the importance of the data smoothing (interpolation) procedure. We use it to reduce random, small-scale, and short-term variations in the original data.

Wave motions are most clearly manifested in the altitude–time dependences of the vertical velocity. As a rule, the velocity changes direction, and the velocity modulus usually increases with altitude. With increasing altitude, the amplitude of wave motions, contributing to variations in vertical velocity, increases. It sometimes reached 12–15 m/s [15]. The study [15] presents data on the delay of wave oscillations of the vertical velocity *V* with increasing altitude, which corresponds to a downward-directed vertical component of the phase velocity. Vertical wavelengths, determined from the altitude profiles *V*(*h*), range from 3–5 km to 30 km.

**Figure 14:**
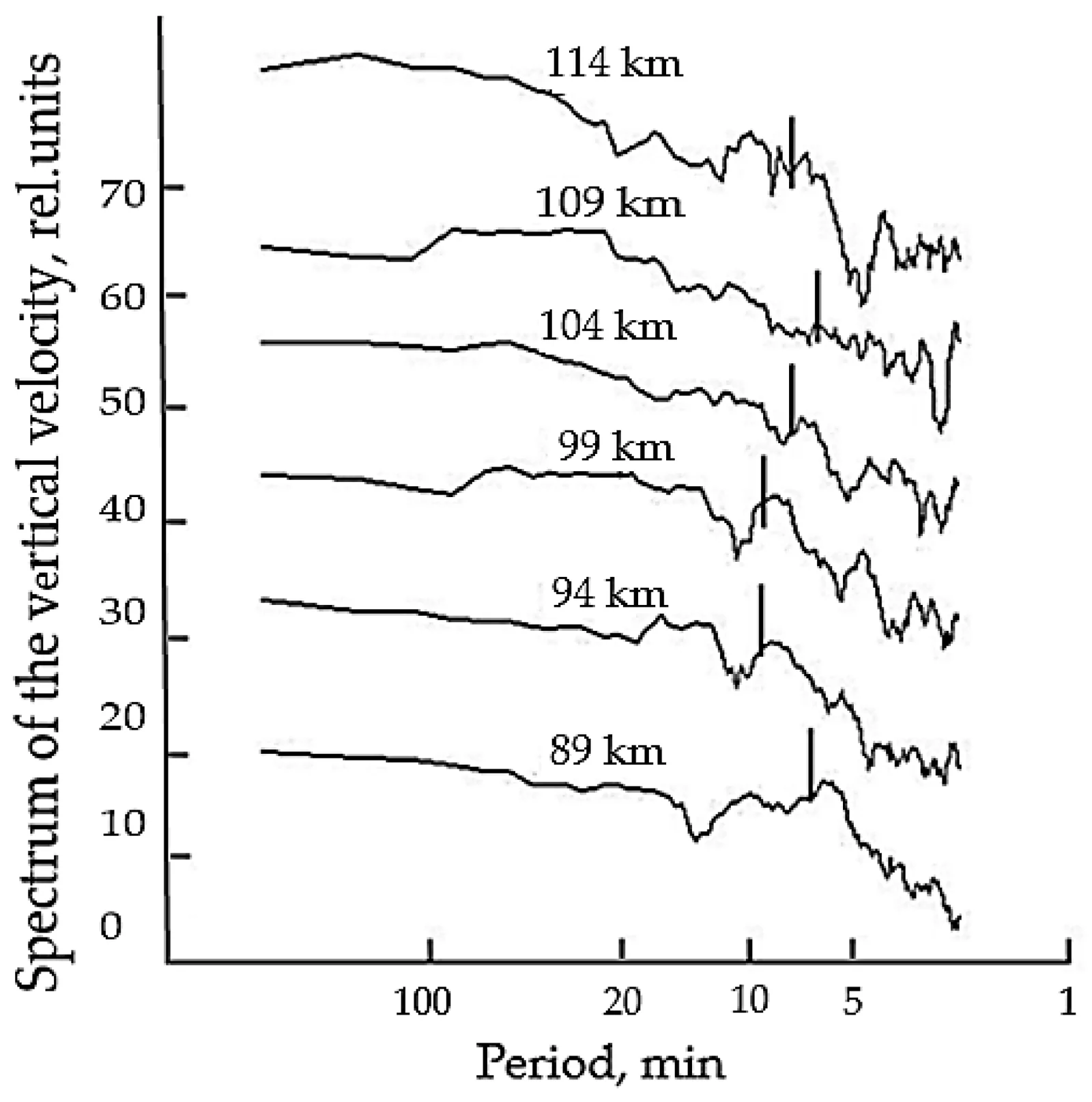
Spectra of the vertical velocity at altitudes from 89 km to 114 km with a step of 5 km for 15 March 2015 (modified version from [15]). The vertical segments mark the Brunt–Väisälä period, which varied in the range of 6.5–7.8 min.

Spectral analysis allows us to identify the predominant frequencies (periods) that make the main contribution to the dynamics of atmospheric parameters. Figure 14 shows characteristic spectra of vertical velocity at mesospheric and lower thermosphere altitudes. The spectra were calculated using the FFT method based on the velocity–time dependence and smoothed using the Hann spectral window [39]. For convenience, the spectra shown in Figure 14 are spaced vertically; therefore, the vertical axis is not shown.

The wave spectra are a power-law function of frequency with an exponent of about 1 and a sharp break near the Brunt–Väisälä frequency (the natural frequency of an air element’s oscillations in the vertical direction), allowing this frequency to be determined experimentally. Based on spectral analysis of vertical velocity variations during different measurement periods, periods of wave motion lasting 5–10, 15, 20, 30–40, and 60 min were reliably identified. These are typical periods of Medium-Scale Traveling Ionospheric Disturbances (MSTIDs) [40]. On some days of particularly long measurements, intense wave motions with periods of 1.5, 2–2.5, 3.3, and more than 4 h were observed. Such periods are characteristic of Large-Scale Traveling Ionospheric Disturbances (LSTIDs) [41].

In Section 4, we demonstrated that the time and height dependence of the vertical and turbulent velocities changes with height and in time. The results strongly suggest that atmospheric waves, including IGWs, are manifested in these variations. This statement is based on our long-term studies of the ionosphere using the API method [15,16,21,22]. We estimated IGW parameters, such as their horizontal wavelength *λ_x_* and period *T_wave_*. For this, we used the dispersion relation for IGWs, the vertical velocity of the environment *V* from our scattered signal phase measurements, and the vertical wavelength *λ_z_* from the vertical velocity altitude profiles [18]. We also used the Brunt–Väisälä frequency (period) data from vertical velocity spectra.

Assuming that the horizontal velocity *V_x_* and the wavelength λ_x_ are much larger than the vertical ones, we can obtain the following estimates

(5) $$\lambda_{x}=\lambda_{z}^{2}\frac{\omega_{g}}{2\pi V_{z}}{;\;T}_{wave}=\lambda_{z}/V\;$$

For *V* = 0.5 m/s, *λ_z_* = 5 km and Brunt–Väisälä frequency *ω_g_* = 1.3 × 10^−2^ Hz (Brunt–Väisälä period *T_g_* = 8 min), we obtained *λ_x_* = 51.7 km and *T_wave_* = 3.5 h. The temporal wave-like variations of the turbopause level and turbulent and regular vertical velocities with a period from 5 min to several hours are typical of IGW. Similar variations in parameters were constantly observed in our experiments [15,16,18,19,20,21,22].

The influence of atmospheric waves on turbulent processes is discussed in most articles containing the results of turbulent parameter measurements. The role of IGWs/AGWs in the excitation and amplification of turbulent phenomena and in variations in the turbopause height is noted [3,9,10,11,13,14,42]. Regarding the influence of geomagnetic disturbances, their direct impact on lower ionospheric turbulence is unclear.

## 6. Conclusions

This paper presents a new method and new results for measuring the turbopause height and turbulent velocity, as well as the velocity of regular vertical motion in the lower ionosphere. All data were obtained using the API method, usually in the autumn season. The API method allows for determining atmospheric turbulence parameters and studying their altitude–temporal variations. The altitude resolution was 150 m or 1.4 km, and the temporal resolution was 15 s, enabling the study of both fast and slow processes in the lower ionosphere.

The turbopause height varies from 85 to 110 km, sometimes descending to mesosphere altitudes. The velocity of turbulent motion (turbulent velocity), averaged over a 5 min interval, is 2.0–2.5 m/s, reaching 5–7 m/s during intense wave activity.

In the region of turbulent mixing of atmospheric gases, the turbulent velocity and the velocity of regular vertical motion are often of the same order of magnitude. Instantaneous vertical velocity varies with altitude and time, typically on a time scale of 15 s and at altitude scales of 1–3 km. The sporadic E-layer enhances the scattered signal in the lower ionosphere. This is a consequence of improved conditions for API formation beneath the E_s_ layer due to the reflection of some of the powerful wave energy from it. Turbulent velocity and the turbopause level are influenced by atmospheric waves with periods ranging from 5 min to several hours.

High variability of data at altitudes of 60–130 km during the day and from day to day, caused by the propagation of atmospheric waves, has been confirmed. Wave-like motions with varying periods are consistently present in the altitude–time variations of the scattered signal amplitude and relaxation time, as well as in the vertical regular and turbulent velocity and the turbopause level. Internal gravity waves (IGWs) are the most likely cause, as variations in these parameters depend on the periods of these waves and MSTID and LSTID.

Measurements of turbopause level and turbulent velocity using the API method generally agree well with results obtained by other known methods. The referenced papers [1,2,3,5,6,7,8,9,10,11,12] provide turbulent velocity values of several meters per second and turbopause heights close to those obtained by the API technique.

In the future, we plan to jointly study variations in turbulent and vertical velocities, as well as neutral atmospheric temperature, measured using the API method. We have accumulated extensive experimental data on this topic. We hope that these will be useful in analyzing the impact of ionospheric variations on GNSS signal delays.

## Figures and Tables

**Table 1:** The range of variation in the turbopause level and the turbulent velocity.

Date	Time Interval, LT	h *h_t_*, km	*V_t_*, m/s	*V*, m/s	<*V*>, m/s	K_p_	W
12 August 2015	17:55–22:35	94–107	0.4–5.8	–0.4; +0.1	–0.9; +1.3	3–4	50
13 August 2015	06:00–09:00	99–106	0.6–5.0	–0.75; +2.15	–0.7; +3.0	2–3	46
27 September 2016	11:30–16:00	90–102	0.1–4.0	–0.8; +0.05	–1.0; +2.0	4–5	9
9 August 2017	16:55–17:55	90–104	0.3–5.3	–0.05; –0.52	–0.7; +0.4	1	5
26 September 2017	12:20–15:40	91–107	0.1–4.0	–0.5; –0.09	–0.65; +0.4	3	10
28 September 2017	16:30–17:50	90–105	0.3–5.0	–0.25; +1.6	–0.56; +1.9	3–4	5
27 September 2018	11:50–16:40	93–104	0.4–4.3	–0.25; –0.15	–0.43; +0.21	2–3	2
28 September 2018	11:30–17:30	91–103	0.1–2.8	–0.22; +0.02	–0.46; +3.2	1	2
26 October 2018	09:15–15:00	88–108	0.1–7.0	–0.38; –0.2	–0.52; +0.33	1–2	2
11 September 2019	12:10–17:10	93–102	0.05–2.3	+0.25; +0.37	+0.16; +0.64	1–2	5

## Data Availability

The data presented in this study are available on request from the corresponding author. The geomagnetic coordinates of the SURA facility are available from https://geomag.bgs.ac.uk/data_service/models_compass/coord_calc.html (accessed on 30 August 2026). Data on solar and geomagnetic activity were taken from the website http://www.wdcb.ru/stp/index.ru.html (accessed on 30 August 2026).

## Abbreviations

The following abbreviations are used in this manuscript:

API	Artificial Periodic Inhomogeneities
AGW	Acoustic Gravity Waves
GNSS	Global Navigation Satellite System
IGW	Internal Gravity Waves
LT	Local Time
MLT	Mesosphere and Lower Thermosphere
MSTID	Medium-Scale Traveling Ionospheric Disturbances (MSTIDs)
LSTID	Large-scale Traveling Ionospheric Disturbances
